# Genotype-Specific Differences between Mouse CNS Stem Cell Lines Expressing Frontotemporal Dementia Mutant or Wild Type Human Tau

**DOI:** 10.1371/journal.pone.0039328

**Published:** 2012-06-18

**Authors:** Miranda E. Orr, Rose Pitstick, Brenda Canine, Karen H. Ashe, George A. Carlson

**Affiliations:** 1 McLaughlin Research Institute, Great Falls, Montana, United States of America; 2 Department of Cell Biology and Neuroscience, Montana State University, Bozeman, Montana, United States of America; 3 Department of Neurology, University of Minnesota, Minneapolis, Minnesota, United States of America; 4 N. Bud Grossman Center for Memory Research and Care, University of Minnesota, Minneapolis, Minnesota, United States of America; Alexander Flemming Biomedical Sciences Research Center, Greece

## Abstract

Stem cell (SC) lines that capture the genetics of disease susceptibility provide new research tools. To assess the utility of mouse central nervous system (CNS) SC-containing neurosphere cultures for studying heritable neurodegenerative disease, we compared neurosphere cultures from transgenic mice that express human tau with the P301L familial frontotemporal dementia (FTD) mutation, rTg(tau_P301L_)4510, with those expressing comparable levels of wild type human tau, rTg(tau_wt_)21221. rTg(tau_P301L_)4510 mice express the human tau_P301L_ variant in their forebrains and display cellular, histological, biochemical and behavioral abnormalities similar to those in human FTD, including age-dependent differences in tau phosphorylation that distinguish them from rTg(tau_wt_)21221 mice. We compared FTD-hallmark tau phosphorylation in neurospheres from rTg(tau_P301L_)4510 mice and from rTg(tau_wt_)21221 mice. The tau genotype-specific phosphorylation patterns in neurospheres mimicked those seen in mice, validating use of neurosphere cultures as models for studying tau phosphorylation. Genotype-specific tau phosphorylation was observed in 35 independent cell lines from individual fetuses; tau in rTg(tau_P301L_)4510 cultures was hypophosphorylated in comparison with rTg(tau_wt_)21221 as was seen in young adult mice. In addition, there were fewer human tau-expressing cells in rTg(tau_P301L_)4510 than in rTg(tau_wt_)21221 cultures. Following differentiation, neuronal filopodia-spine density was slightly greater in rTg(tau_P301L_)4510 than rTg(tau_wt_)21221 and control cultures. Together with the recapitulation of genotype-specific phosphorylation patterns, the observation that neurosphere lines maintained their cell line-specific-differences and retained SC characteristics over several passages supports the utility of SC cultures as surrogates for analysis of cellular disease mechanisms.

## Introduction

The ability to generate human embryonic stem cell lines by somatic cell nuclear transfer [1] or to produce induced pluripotent stem cells by reprogramming [2] provides the opportunity to capture the genetics of diseased patients. The availability of patient-specific SC lines offers the possibility of transplantation for cell replacement or the delivery of therapeutic agents, and patient-tailored drug therapy. Use of disease-specific SC lines to dissect cellular disease processes is a burgeoning field yielding promising results [3]–[16].

While our goals are to develop and validate approaches that can be applied to patient-specific cell lines, mouse models offer important advantages for experimental analysis. Each human patient is unique, but members of inbred mouse strains are genetically homogeneous, allowing discrimination of variation that may be inherent to SC isolation from genetic effects. Mouse models also allow tracking of the subtle biochemical, histological, and behavioral changes that occur long before clinical signs appear. By exploiting SC lines from well-characterized mouse models, we hope to relate cell culture phenotypes to pre-clinical pathogenic events.

Frontotemporal dementia (FTD) is a neurodegenerative disorder in which aggregates comprised of microtubule associated protein tau (MAPT) form in neurons. FTD, like other tauopathies, including Alzheimer’s disease, is characterized by tau phosphorylation and aggregation events associated with neuronal death and dementia. Transgenic mouse lines expressing human MAPT with a proline to leucine mutation at amino acid 301 (P301L) recapitulate aspects of familial FTD [17]–[20]. Ashe and colleagues [19], [20] developed a regulatable bigenic transgenic line rTg(tau_P301L_)4510 (hereafter, rTg(tau_P301L_) is used to indicate rTg(tau_P301L_)4510) in which MAPT transgene expression is largely restricted to forebrain cells to avoid early spinal cord pathology that develops in mice with prion protein promoter driven mutant tau [18]. MAPT transgene expression can be suppressed with doxycycline.

Here we report the isolation and characterization of neurosphere lines from rTg(tau_P301L_) mice and from recently created transgenic mice that express comparable levels of human tau_wt_, rTg(tau_wt_)21221, hereafter referred to as rTg(tau_wt_) [21]. Production of neurospheres is a well-established technique and these multi-cellular aggregates consist of CNS-SCs, lineage-committed, and differentiated cells [22]–[25]. The effects of genetics on cell proliferation, differentiation, and mature cell types can be assessed in neurosphere cultures [22], [23]. We evaluated the effects of the P301L mutation on tau phosphorylation in mice and in SC lines derived from them. Neurospheres recapitulated the genotype-specific differences in tau phosphorylation seen in mice, and we found genotype-dependent differences in the fraction of transgene expressing cells, the level of phosphorylation, and in filopodia-spine densities.

## Materials and Methods

### Mice

rTg(tau_P301L_) and rTg(tau_wt_) mice, along with similar lines described in Results, were generated using a bigenic system of responder and activator transgenes. Tg(tau_P301L_) and Tg(tau_wt_) mice (designated TRE-tau_P301L­_ and TRE-tau_wt_) carry their corresponding tetO-tau responsive element transgenes and were produced and maintained on the FVB/NCr background [19], [21]. Tg(CK-tTA) mice that express a Camk2α-driven tet transactivator transgene have been described previously and are congenic on a 129S6 genetic background [19], [26]. The TRE-tau_wt_ construct was identical to that used to construct Tg(tau_P301L_)4510 mice except for the presence of a wild type proline codon at position 301. The rTg(tau_wt_)21221 line expressed human tau at levels comparable to rTg(tau_P301L_)4510 [21]. All rTg mice and their controls, which expressed either the CK-tTA or TRE-tau transgene alone, were genetically homogeneous (FVB × 129)F1 hybrid mice.

Mapt^tm1(GFP)Klt^ Tg(MAPT)8cPdav/J mice, referred to as 8c *Mapt^0/0^*, have a targeted disruption of mouse *Mapt* exon one and express a complete human MAPT transgene. They have been described previously [27], [28]. Breeder pairs of mixed genetic background were obtained from the Jackson Laboratory. For these experiments, we bred out the 8c human tau transgene to produce mice lacking endogenous mouse tau. These mixed background *Mapt^0/0^* mice were mated with Tg(tau_wt_), Tg(tau_P301L_), or Tg(CK-tTA) mice. Mice hemizygous for activator or responder transgenes and heterozygous for endogenous *Mapt* were backcrossed to the *Mapt^0/0^* line to produce Tg(tau_wt_) *Mapt^0/0^*, Tg(tau_P301L_) *Mapt^0/0^*, and Tg(CK-tTA) *Mapt^0/0^* mouse lines on mixed genetic backgrounds that were used to produce *Mapt^0/0^* rTg mice with both the responder and transactivator transgenes.

### Ethics Statement

The studies at McLaughlin Research Institute (MRI), which is fully accredited by AAALAC International, were carried out in accordance with the Guide for the Care and Use of Laboratory Animals of the National Institutes of Health, U.S. Public Health Service. MRI’s Animal Assurance number from the Office of Laboratory Welfare of the National Institutes of Health is A3901–01. All procedures involving animals were reviewed and approved by MRI’s Institutional Animal Care and Use committee under protocol GAC-05. Fetuses were harvested from timed pregnant females euthanized by cervical dislocation performed by trained personnel.

### Generation, Maintenance, and Differentiation of Neurosphere Lines

TRE-tau_wt_ or TRE-tau_P301L_ females were mated with Tg(CK-tTA) males. Neurospheres were isolated from whole brains from individual E14 fetuses using established protocols described previously [22], [29]. The cells were grown in serum-free “Complete” NeuroCult NSC Proliferation Medium comprised of NeuroCult NSC Basal Medium (Stem Cell Technologies (SCT)) supplemented with Proliferation Supplement (SCT), 20 ng/mL rhEGF (SCT), and penicillin/streptomycin (GIBCO). The neurosphere sex was determined by PCR genotyping for the X and Y chromosome genes *Smcx* and *Smcy* [30]. For passage, neurospheres were enzymatically dissociated to single cells using the Papain Tissue Dissociation Kit as per the manufacturer’s instructions (Worthington Biochemical Corporation). Dissociated cells were seeded at a concentration of 10^5^ cells/mL in 15 mLs “Complete” NeuroCult NSC medium (Stem Cell Technologies) and maintained in a humidified incubator at 37°C in 6% O_2_ and 6.8% CO_2_ (balance N2). Neurosphere lines were passaged every 5–7 days. Time of passage was determined by neurosphere density and media color change. Colorimetric cell proliferation assays (MTT) to assess neurosphere growth rates were conducted according to the manufacturer’s recommendations (Cell Growth Determination Kit, Sigma).

For immunofluorescent analysis (IFA) or immunohistochemistry, undifferentiated single cells, 8×10^4^ cells/well, were plated on fibronectin (Invitrogen) coated 24-well glass bottom plates (Greiner) and placed in a humidified incubator at 37°C at 6% CO_2_ 6% O_2_ overnight.

To induce differentiation, dissociated cells were plated on laminin/poly-L-ornithine (15 µg/mL poly-L-ornithine and 5 µg/mL laminin) 24-well coated glass plates at a density of 12×10^4^ cells/well in complete NeuroCult medium (SCT). After overnight culture the growth factor-containing medium was removed and 0.3 µmol retinoic acid was added every other day for seven days. Between days 8–25, cells were fed with B27-containing medium (Gibco) on alternate days. IFA was performed on day 21 or day 25.

### Immunoblots

Adult mouse hemibrains for Western blot analysis were prepared as described previously [19]. Mouse brains or neurospheres were homogenized in ice-cold buffer consisting of 10mM Tris-HCl, 1mM EGTA, 0.8M NaCl, 10% sucrose, pH 7.4, Complete Protease Inhibitor Cocktail (Roche), and Phosphatase Inhibitor Cocktail II (Calbiochem). 20 µg protein equivalent from adult brain homogenates, 3×10^6^ cells from neurosphere lysates, or 10 µL from a 10% embryonic brain homogenate were electrophoresed on 12% Bis-Tris gels (Invitrogen) and electro-blotted onto PVDF membranes (Millipore). In some cases, neurosphere samples were treated with phosphatase prior to running; protein lysates were suspended in 0.5 µg/10 µL of NEBuffer 3 (New England Biolabs) and incubated with 10U/µL of calf intestinal phosphatase (CIP) (New England Biolabs) for 60 minutes at 37°C. We used the following anti-tau antibodies: Tau13 at 1∶5000 (Covance); Tau1 at 1∶200 (Chemicon); CP13, AT8, PHF-1, and DA9 (provided courtesy of Dr. Peter Davies) at 1∶200. The specificity of these antibodies is summarized in Table 1. Loading and transfer control anti-GAPDH was used at 1∶2000 (Chemicon). Goat anti-mouse HRP-conjugated IgG (Biorad) secondary antibody was diluted 1∶10,000. SuperSignal West Pico reagent (Pierce) was used for membranes probed with Tau13, Tau1, PHF-1, and DA9; the ECL Plus substrate (Amersham) was used for membranes probed with phospho-tau antibodies CP13 and AT8 and to detect immunoreactivity. For both chemiluminesence systems, membranes were incubated with substrate for 3 minutes. Chemiluminescence was detected using a Biorad VersaDoc at 3–5 minute exposures. Tau levels were normalized to GAPDH levels to correct for loading and transfer differences.

**Table 1:** Antibodies: epitopes recognized and relative sizes of mutant and wild type human tau in neurospheres and mice.

	Relative Size		
Antibody	E14 Mice	Neurospheres	2.5-mo old mice
Tau13 (human residues 2–18)	tau_wt_>tau_P301L_	tau_wt_>tau_P301L_	tau_wt_>tau_P301L_
CP13 (pSer^202^)	tau_wt_>tau_P301L_	tau_wt_>tau_P301L_	tau_wt_>tau_P301L_
AT8 (pSer^202^/pThr^205^)	tau_wt_>tau_P301L_	tau_wt_>tau_P301L_	tau_wt_>tau_P301L_
PHF-1 (pSer^396^/pSer^404^)	tau_wt_>tau_P301L_	tau_wt_>tau_P301L_	tau_wt_>tau_P301L_
Tau1 (non-phospho Ser^198^/Ser^202^)	tau_wt_ = tau_P301L_	tau_wt_ = tau_P301L_	tau_wt_>tau_P301L_

Epitopes recognized by each antibody are shown in parentheses. The more slowly migrating, and hence higher apparent MW, band is to the left and separated from the smaller, more quickly migrating tau species by the symbol “>”. The “ = ” symbol indicates no difference in apparent size of the tau species.

### Immunofluorescence

For whole neurosphere IFA, neurospheres were fixed in 4% phosphate buffered paraformaldehyde followed by 30% sucrose and equilibrated at 4°C overnight. Fixed neurospheres were placed in embedding medium (Sakura Tissue-Tek O.C.T.) and snap frozen in liquid nitrogen. Neurospheres were sectioned at −15°C; 8 µm sections were adhered to SuperFrost Plus microscope slides (Fisher) and stored at −20°C for later IFA. Cryosectioned neurospheres were stained with the following primary antibodies: rabbit anti-Nestin at 1∶200 (Covance); mouse anti-human Tau13 at 1∶5000 (Covance); mouse anti-Tau46 at 1∶600 (Cell Signaling). The secondary antibodies Alexa488-goat anti-mouse and Alexa546-goat anti-rabbit were used at 1∶1500 dilution (Molecular Probes).

For analysis of individual undifferentiated cells plated on fibronectin substrate and differentiated cells plated on LPO substrate, cells were fixed at 37°C in PIPES fixative (0.1 M PIPES, 1.0 mM EGTA, 3mM MgSO_4_ and 3% PFA). Cells were permeabilized in 0.3% Triton X-100, placed in blocking buffer (5% normal goat serum, 5% glycerol, and 0.04% sodium azide in PBS), then incubated in primary antibody. Primary antibodies Tau13, Tau46, Tau1, CP13, AT8, PHF-1, and DA9 were used at dilutions described above. Rabbit anti-β tubulin III at 1∶2000 (Covance); chicken anti-MAP2 at 1∶10,000 (Covance); and rabbit anti-GFAP at 1∶1000 (Chemicon) also were used. The following secondary antibodies from Molecular Probes were diluted 1∶1500: goat anti-mouse Alexa Fluor 546; goat anti-mouse Alexa Fluor 488; goat anti-rabbit Alexa Fluor 546; goat anti-rabbit Alexa Fluor 488; goat anti-rabbit Alexa Fluor 647. The goat anti-chicken Alexa Fluor 488 antibody was from Jackson ImmunoResearch, Inc and used at 1∶200. Cells were coverslipped with ProLong Gold antifade reagent containing DAPI (Invitrogen). Images were acquired with a Nikon Eclipse TE2000 microscope and analyzed with MetaMorph software. The proportion of total cells expressing human tau (Tau13 positive) was determined for each cell line. Fluorescence intensity and area stained, the threshold parameters, were based on Tg(CK-tTA) control cell fluorescence and fluorescence of cells incubated only with secondary antibody. All image acquisition and cell counts were conducted by an experimenter blind to genotype.

### Immunohistochemistry

Cryosectioned neurospheres were thawed overnight and the Vector M.O.M. peroxidase immunodetection kit was used to detect transgene expression (Vector Laboratories, Inc) per manufacturers instructions. Neurospheres were probed with Tau13 at a dilution of 1∶5000.

### Filopodia-Spine Counts

To determine filopodia-spines densities, total Map2 positive neurite lengths were measured 21 or 25 days post-differentiation; an individual not involved in this experiment assigned a code to each slide so the experimenter was blind to genotype. All Map2-positive projections were measured using MetaMorph software and counted along the Map2 positive neurites; projections >12 µm were called neurites and <12 µm were called filopodia-spines. The number of filopodia-spines/100 µm was calculated. Between 75–100 total Map2 and TUJ-1 double positive cells were counted from each cell line. The average filopodia-spine density/cell was calculated for each cell line.

### Statistical Analyses

Both ANOVA and paired t tests were performed using StatView 5 for Macintosh (SAS Institute, Inc.) for the determining significance levels for differences in tau expression levels between rTg(tau_wt_) and rTg(tau_P301L_) neurosphere lines. For paired analysis, individual rTg(tau_wt_) and rTg(tau_P301L_) were paired within each experiment according to order of harvest (line number); in two experiments the numbers of mutant and wild type lines were not equal, reducing the number of samples from 22 to 20. For the ANOVA, experiment and tau genotype were independent variables with fraction human tau positive, the dependent variable. For ANOVA of the effects of differentiation on the fractions tau positive cells in rTg(tau_P301L_) and rTg(tau_wt_) neurosphere lines, differentiation state and tau genotype were the independent variables. Independent variables for analysis of spine density were time post-differentiation and genotype. Post-hoc Bonferroni/Dunn analysis was performed on ANOVA results. Correlation coefficients for the relationship between spine density and fluorescence intensity were calculated using StatPlus.

## Results

### CK-tTA Driven TRE-tau Transgenes were Expressed in Neurospheres and Fetal Mice

We previously generated a Tg(tau_wt_) transgenic line that harbors human wild type 4R0N tau transgenes driven by the same TRE as Tg(tau_P301L_). When crossed to Tg(CK-tTA) mice, the resulting rTg(tau_wt_) mice express human wild type tau at levels comparable to those in rTg(tau_P301L_) mice [21]. We refer to mice and cultures that carried both responder and activator transgenes as rTg, and refer to those that carried only the activator or responder transgene as Tg(CK-tTA) or TRE-tau_P301L_ and TRE-tau_wt_ controls. Activator and responder mouse lines were maintained hemizygous for their respective transgenes; therefore one-fourth of all pups were expected to contain both the activator and responder transgenes and express the protein of interest.

**Figure 1:**
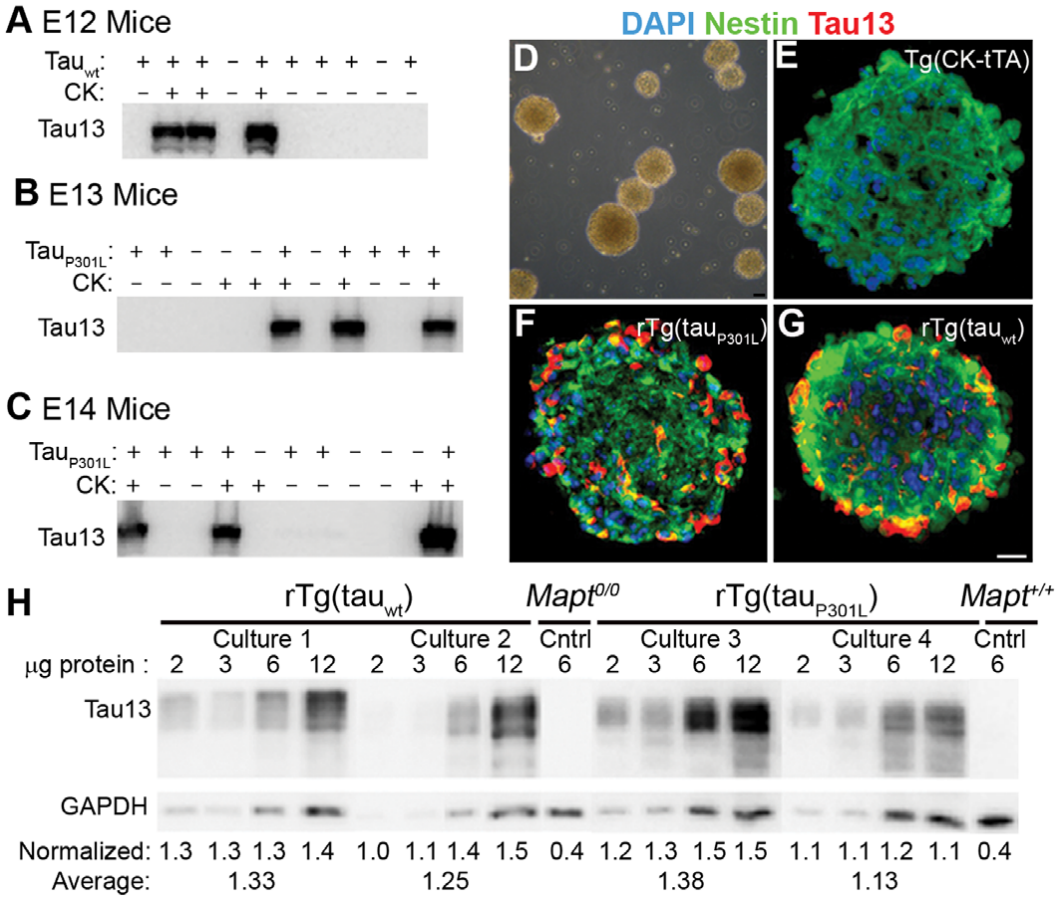
rTg(tau_wt_) and rTg(tau_P301L_) fetuses and neurospheres expressed transgene encoded human tau. (A–C) Brains were taken from Tg(CK-tTA) × TRE-tau pups at E12 (A) E13 (B) and E14 (C) for WB analysis with the human specific anti-tau antibody, Tau13. We saw immunoreactivity only in brain homogenates from pups that genotyped positive for both transgenes. None of the samples from pups with either the transactivator or responder transgene alone or with neither transgene were immunoreactive with Tau13. (D–G) Neurosphere cultures generated from E14 Tg(CK-tTA) × Tg(tau) mouse litters grew as non-adherent neurospheres and expressed human tau. (D) Representative phase contrast image of neurospheres in culture. (E–G) Fixed and cryosectioned neurospheres analyzed by fluorescence microscopy demonstrated that nearly all cells in Tg(CK-tTA) (E), rTg(tau_P301L_) (F), and rTg(tau_wt_) (G) expressed the CNS-SC protein, nestin (green). rTg(tau_P301L_) (F) and rTg(tau_wt_) (G) neurospheres contained cells that were strongly immunoreactive with Tau13 (red); control Tg(CK-tTA) (E) neurospheres did not. DAPI nuclear stain is blue in E–G. (H) Western blot analysis from two independent rTg(tau_wt_) neurosphere cell lines (cultures 1 and 2) and two independent rTg(tau_P301L_) neurosphere cell lines (cultures 3 and 4) showed comparable levels of human tau between genotypes. Tau13 immunoreactivity was normalized to GAPDH. The normalized values indicated that human tau levels were comparable between genotypes. Lysates from cultures 1 and 2 were probed together on one blot, and lysates from cultures 3 and 4 were probed together on another. Control samples expressing only endogenous mouse tau (*Mapt^+/+^*), or neither mouse nor human tau (*Mapt^0/0^*) were included on each membrane; only one set is shown here for the sake of simplicity.

Expression of *Camk2α*, the promoter driving tTA expression, is largely restricted to forebrain neurons [31]. CK mRNA [32] and CK-tTA-driven tau_P301L_ expression [20] had been reported to be absent in mice until postnatal day 3. In contrast, our Western blot analysis showed CK-driven expression of human tau_wt_ and human tau_P301L_ as early as embryonic day 12 (E12) (Figure 1A–C). This verified transgene expression prior to E14, the developmental age that neurospheres are harvested.

### rTg(tau_wt_) and rTg(tau_P301L_) Neurospheres Expressed Human Tau at Comparable Levels

Neurosphere cultures were generated from individual fetuses from Tg(CK-tTA) × TRE-tau_wt_ and Tg(CK-tTA) × TRE-tau_P301L_ matings, and were genotyped. All cultures grew as non-adherent free-floating aggregates characteristic of neurospheres (Figure 1D). Neurospheres were probed with antibody for nestin, an intermediate filament protein expressed by CNS-SCs, and were tested for human tau protein expression by IFA and Western blot with the Tau13 antibody. Nearly all cells in neurospheres were nestin-positive (Figure 1E–G) as previously reported for neurospheres from other mouse strains (28). Neurospheres from rTg(tau_P301L_) and rTg(tau_wt_) cultures contained cells that also expressed human tau (Figure 1F–G), but Tg(CK-tTA) controls did not (Figure 1E). Western blot analyses on neurosphere lysates confirmed human tau expression in rTg(tau_P301L_) and rTg(tau_wt_) neurospheres, and showed that, as in mice, transgenic human tau was expressed at comparable levels in neurospheres derived from each genotype (Figure 1H).

### Variation in Tau Expression among individual Neurospheres and Independent Neurosphere Lines

**Figure 2:**
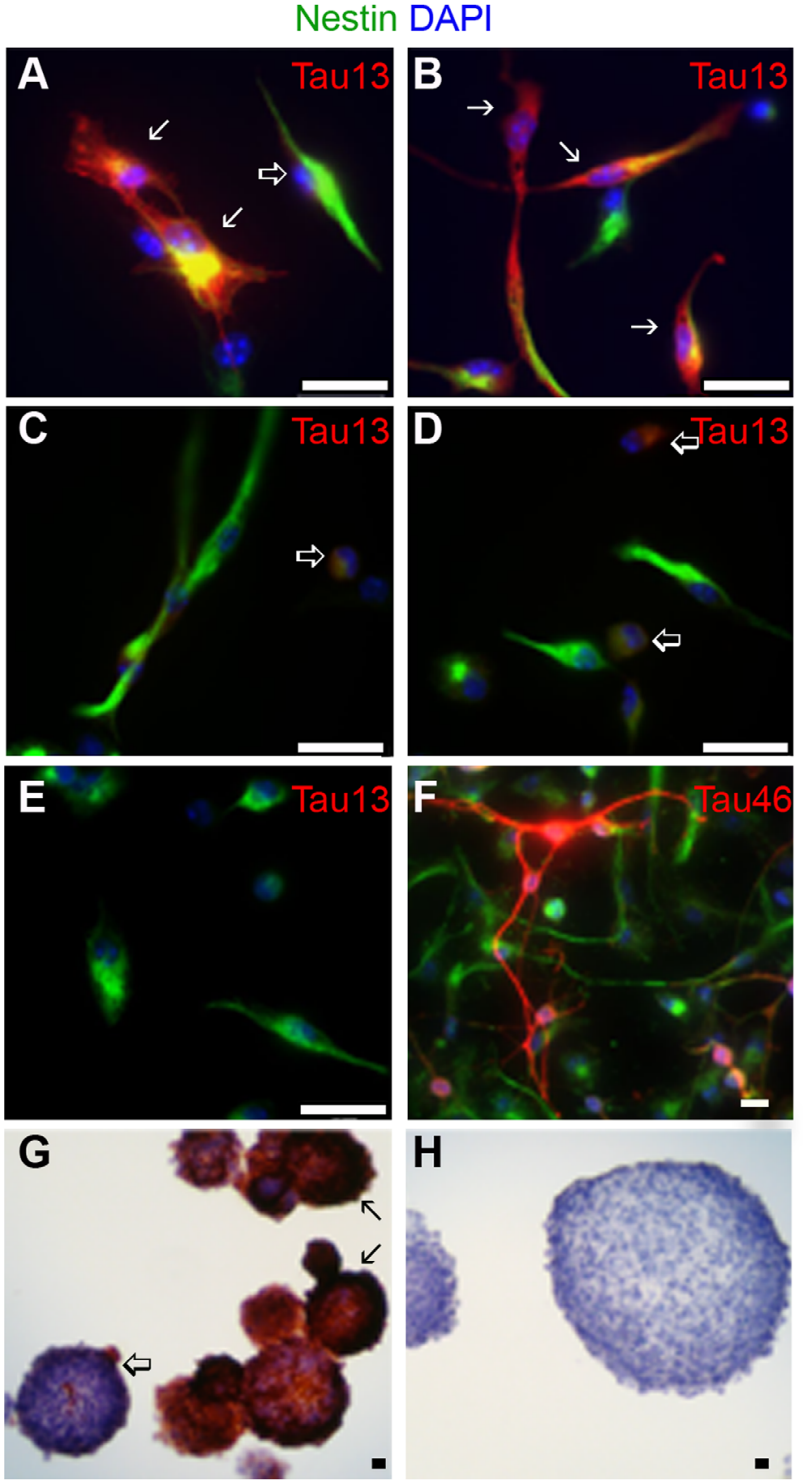
Single-cell staining revealed protein expression heterogeneity in transgene expressing cells. Nestin staining is shown in green (A through F), Tau13 (A through E) or Tau46 (F) in red, and DAPI in blue (A through F). A proportion of cells from rTg(tau_wt_) (21–30%) (A) and rTg(tau_P301L_) (38–44%) (B) cultures displayed strong immunoreactivity with Tau13 (solid arrow), while others had weak immunoreactivity with Tau13 (open arrow). Cultures containing responder transgene alone, TRE-tau_wt_ (C) and TRE-tau_P301L_ (D), did not contain any cells with strong Tau13 immunoreactivity, but they did contain some cells with weak immunoreactivity (open arrows) indicative of leaky transgene expression. (E) Cultures containing only the transactivator transgene, Tg(CK-tTA), did not express human tau but were immunoreactive with Tau46 (F), an anti-tau antibody that reacts with both mouse and human tau. Nearly all cells of all genotypes were immunoreactive with the anti-nestin antibody (green). Scale bar  = 25 µm. (G) Immunohistochemistry of cryosectioned neurospheres probed with Tau13 revealed that some neurospheres from rTg cultures, rTg(tau_P301L_) shown here, contained a large proportion of transgene expressing cells (closed arrow), other neurospheres from the same culture contained very few transgene expressing cells (open arrow). (H) Control Tg(CK-tTA) neurospheres were not immunoreactive with Tau13.

Individual neurospheres within a single cell line (derived from a single fetal brain) varied in transgene expression. Cryosectioned neurospheres stained with Tau13 indicated that transgene-expressing cells were not preferentially located at the periphery nor deep within most spheres (Figure 1F, G; 2G), but neurospheres varied in transgene expression within a single culture. Some neurospheres in a culture contained nearly 100% tau transgene expressing cells, whereas other neurospheres from the same culture contained few, if any, human tau-expressing cells (Figure 2G).

We analyzed individual cells for human tau protein levels by measuring Tau13 fluorescence intensity. At passages 3 and 6 we dissociated, fixed, and immunostained neurospheres (Figure 2). Total cell numbers were based on intermediate filament nestin and nuclear DAPI stains. The fraction of cells that expressed human tau was determined. A cell was considered positive for transgene expression if its Tau13 fluorescence was greater than the background fluorescence seen when incubated only with secondary antibody. Figure 2A and 2B show cells strongly positive with Tau13 in rTg cultures. Figures 2C and 2D indicate cells with very low level expression of human tau in TRE cultures carrying the responder transgene, but lacking the transactivator transgene; such minimally reactive cells were not seen in cultures positive only for the transactivator transgene (Figure 2E and 2H).

**Figure 3:**
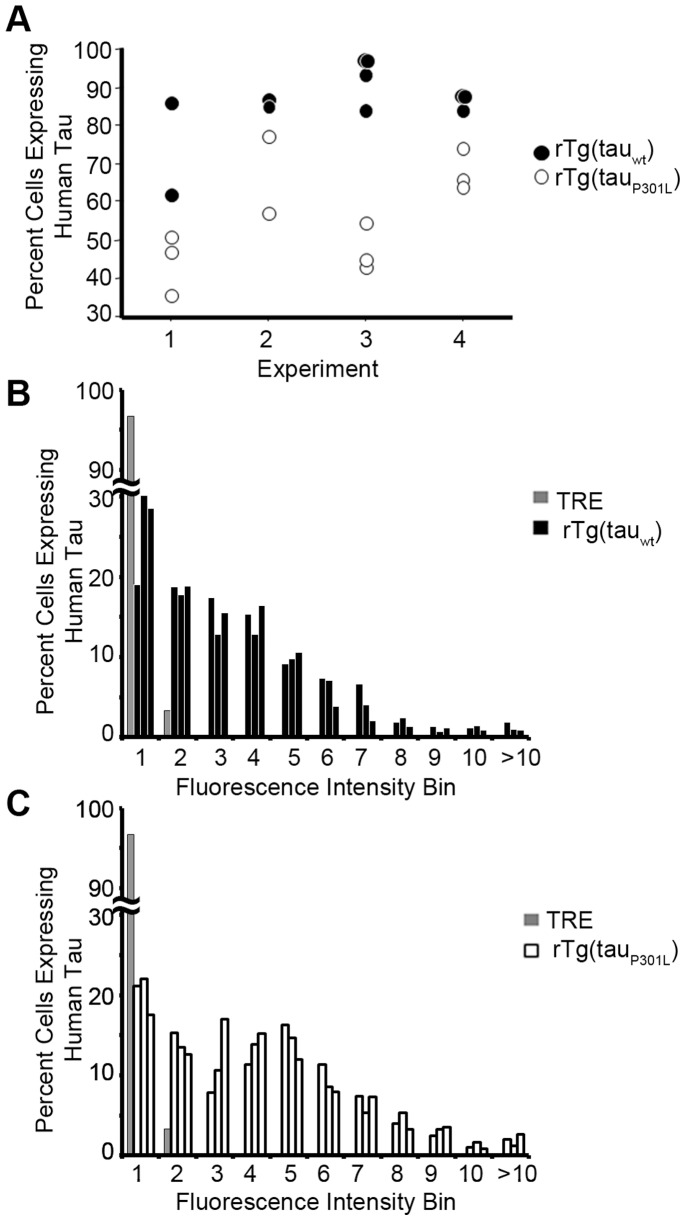
rTg(tau_wt_) neurospheres contain more human tau expressing cells than rTg(tau_P301L_) neurospheres. Undifferentiated single cells (shown in Figure 2) stained with DAPI and nestin were counted and the proportion co-expressing Tau13 was determined. (A) Consistently the proportion of cells co-expressing Tau13 was significantly higher in rTg(tau_wt_) cultures than rTg(tau_P301L_) cultures. Four independent experiments consisting of 11 independent rTg(tau_wt_) cultures and 11 independent rTg(tau_P301L_) cultures are shown here (ANOVA: variation due to experiment F  = 5.5, P = .01; ANOVA mutant versus wild type F  = 65.9, P<0.0001. Interaction between experiment and tau genotype was marginally significant: F  = 3.8, P  = 0.035.) Following Bonferonni/Dunn correction, the mutant versus wild type comparison remained significant at P<0.0001. Paired t test also indicated a significant difference between rTg(tau_P301L_) and rTg(tau_wt_) neurospheres (P<0.0001). Closed circles represent tau_wt_; open circles represent tau_P301L_. (B, C) Fluorescence intensity histograms from Figure 3A’s Experiment 3 cells revealed heterogeneity in transgene expressing cells. Transgene positive cells were binned (1 to >10) based on fluorescence intensity. “1” indicates a “dim” cell with low transgene expression (open arrow from Figure 2), and bins 6 through >10 indicated “bright” cells with high transgene expression (closed arrow from Figure 2); proportion of cells is on the y-axis. The left-skewed rTg(tau_wt_) histogram (B) indicated that most Tau13-positive cells expressed human tau at low levels. In contrast, the bimodal rTg(tau_P301L_) histogram indicated two distinct cell populations: one comparable to rTg(tau_wt_) and one with higher transgene expression levels. TRE cell lines, TRE-tau_P301L_ in this experiment, expressed human tau at low levels; all cells fell in bins 1 and 2.

Cell lines generated from rTg(tau_wt_) fetuses contained a higher proportion of cells expressing human tau than cultures harvested from rTg(tau_P301L_) fetuses (Figure 3A). This was consistent among four independent experiments (p<0.0001 ANOVA). Each experiment was comprised of 2–4 independent rTg cell lines each produced from an individual fetus in litters harvested at the same time from Tg(CK-tTA) × TRE-tau_wt_ and Tg(CK-tTA) × TRE-tau_P301L_ matings. The percentages of cells expressing tau_P301L_ was less in each experiment than the percentages of cells expressing tau_wt_, though comparison of lines derived from fetuses harvested at different times showed some overlap between mutant and wild type lines. The fraction of positive cells within each culture persisted over passage.

To further assess effects of mutant and wild type human tau on the fraction of tau positive cells, we generated neurospheres from three additional transgenic mouse lines that express the same human tau variants as rTg(tau_P301L_)4510 and rTg(tau_wt_)21221, but at lower levels as assessed by immunoblotting. In a single experiment, we determined the percent of human tau expressing cells present in neurosphere cultures produced from the additional mouse lines. rTg(tau_wt_)14238 and rTg(tau_P301L_)14718 mice both express human tau at ∼50% the level of rTg(tau_P301L_)4510 and rTg(tau_wt_)21221. The single neurosphere culture derived from a rTg(tau_wt_)14238 fetus expressed human tau in 42.5% of its cells, while the three neurosphere cultures derived from rTg(tau_P301L_)14718 fetuses had 46%, 45%, and 71% expressing cells. The third transgenic mouse line, rTg(tau_P301L_)14319, expressed human tau at approximately 75% the level of rTg(tau_P301L_)4510 and expressed human tau in ∼60% of its cells. In general, the proportions of cells expressing human tau in the additional lines were within the range seen in the larger series of experiments and reinforce the observation that the proportion of cells expressing tau in an individual line is not necessarily predictive of human tau genotype.

To determine the profile of Tau13 fluorescence intensity of transgene expression, transgene-positive cells were binned (1 to >10) based on fluorescence intensity (Figure 3B). The “dim” cells indicated by the open arrows in Figure 2C and 2D fall in bin 1, while the bright cells in Figure 2A and 2B are in bins 6 to >10. Fluorescence intensity histograms are shown for three independent rTg(tau_wt_) cultures (Figure 3B) and for three independent rTg(tau_P301L_) cultures (Figure 3C) taken from one experiment (Experiment 3 from Figure 3A). While the rTg(tau_P301L_) cultures had a lower proportion of total cells expressing human tau (Figure 3A), they contained a greater proportion of cells that expressed higher levels of tau (brighter fluorescence intensity) (Figure 3C) than rTg(tau_wt_) cells (Figure 3B). The TRE cultures, which expressed the responder transgene alone, contained a fraction of cells, approximately 41% of Tg(tau_P301L_) cells and 19% of Tg(tau_wt_) cells that expressed human tau at very low levels consistent with the leaky expression reported previously [19], [33].

### Tau Phosphorylation in Neurospheres

**Figure 4:**
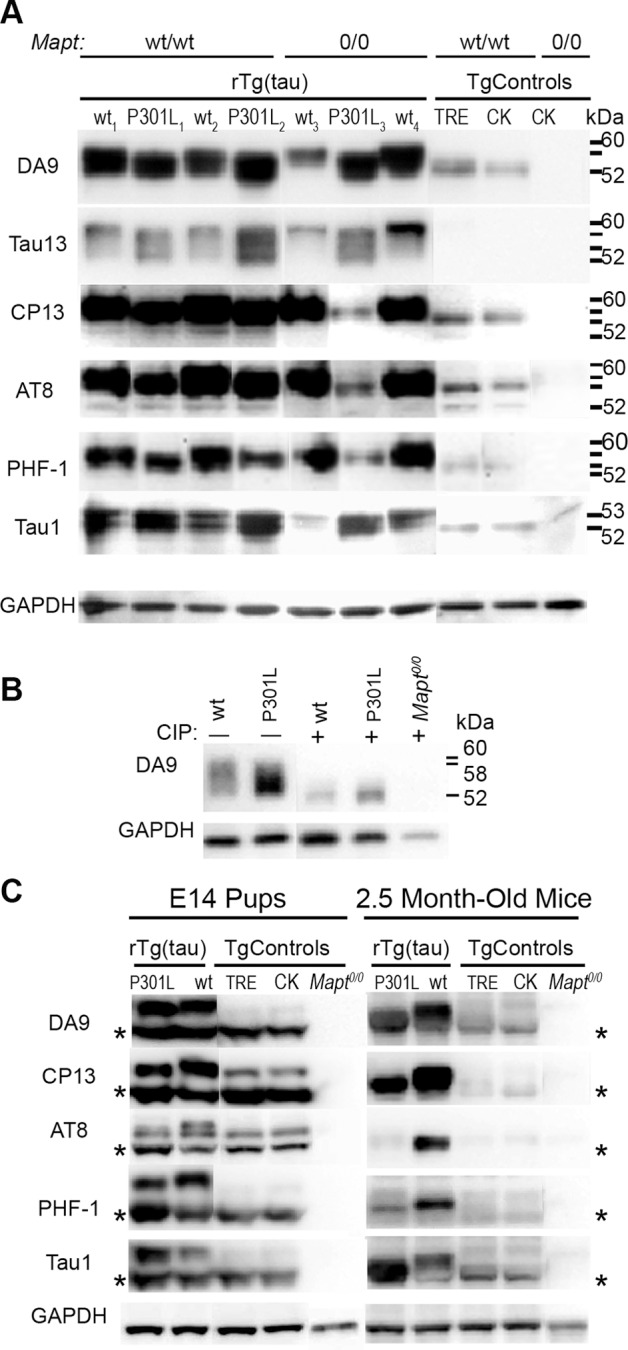
Transgene-encoded human tau_wt_ is more heavily phosphorylated than tau_P301L_. (A) Electrophoresed and blotted neurosphere lysates were probed with antibodies against total tau (mouse and human): DA9; human tau: Tau13; phospho-tau: CP13, AT8, and PHF-1; and non-phospho tau: Tau1. In control samples, TRE (tau_P301L_ shown here) and Tg(CK-tTA), all antibodies, except Tau13, revealed a mouse tau band at ∼52kDa that is absent from *Mapt^0/0^* samples. Human tau from rTg samples migrated as a diffuse series of bands indicative of a heterogenous population of tau species. Human tau_wt_-expressing cells contained more slowly migrating tau species (∼53 to 60 kDa) than tau_P301L_-expressing cells (∼53 to 58 kDa) with all anti-tau antibodies, except Tau1, indicating a more heavily phosphorylated tau species in rTg(tau_wt_) than rTg(tau_P301L_). Tau1 probed samples revealed an identical migration pattern with both rTg genotypes. In all blots, MW lines indicate 60, 58, 53, 52 kDa, respectively. All samples probed with the each tau antibody were blotted on the same membrane. GAPDH was probed simultaneously with each tau antibody; the example of GAPDH immunostaining shown is taken from the Tau1 blot. *Mapt^0/0^* and control samples were run on each blot but rearranged in the figure for presentation purposes. See Figures S1 for the original blots for each antibody showing the *Mapt^0/0^* and control samples in their locations before rearrangement for presentation, along with GAPDH immunostaining for each blot. (B) Tau protein from rTg(tau_wt_) *Mapt^0/0^* and rTg(tau_P301L_) *Mapt^0/0^* neurosphere lysates had the same electrophoretic mobility after phosphatase treatment. Untreated samples (-) showed the characteristic slower migrating (∼60 kDa) trailing edge for tau_wt_ compared to tau_P301L_. Calf-intestinal phosphatase (CIP) treatment (+) abolished the higher molecular weight phospho-tau bands (>52 kDa) leaving a single tau band migrating at ∼52kDa in both rTg genotypes. (C) Human tau from rTg(tau_wt_) E14 and adult mice contained more slowly migrating phospho-tau species than rTg(tau_P301L_) as seen in neurospheres. No immunoreactivity was observed in *Mapt^0/0^* samples with any anti-tau antibody; *  =  mouse tau. All samples probed with each tau antibody were blotted on the same membrane. GAPDH was probed simultaneously with each tau antibody; the example of GAPDH immunostaining shown is taken from the AT8 blot. *Mapt^0/0^* and control samples were run on each blot but rearranged in the figure for presentation purposes. See Figures S2 and S3 for complete, un-rearranged blots showing all samples that were run.

A characteristic of both tauopathy patients and the rTg(tau_P301L_)4510 FTD mouse model is tau misprocessing, primarily phosphorylation. To determine if mutant tau-specific phosphorylation occurred in neurospheres, we used a battery of phospho-tau specific antibodies. Due to the numerous sites for phosphorylation and other posttranslational modifications of the tau protein, immunoblotted tau most often does not resolve as distinct bands but instead as a heterogeneous protein smear. To discriminate distinct tau species within the total tau population, we used antibodies specific to total tau (DA9), human tau (Tau13), and phospho-tau epitopes (CP13, AT8, PHF-1, Tau1; see Table 1 for epitope specificity). Each antibody was probed on independent membranes simultaneous with GAPDH; the GAPDH blot presented in Figure 4 is from the Tau1 blot. Complete blots for each antibody are shown in Figures S1, S2, S3.

Immunoblotting with DA9 allowed us to determine total tau (mouse and human) expression and visualize the electrophoretic migration pattern of tau from both species (Figure 4A.) Densitometric analysis with DA9 indicated that rTg neurospheres expressed over five-times the amount of total (mouse and human) tau protein than Tg(CK-tTA) control samples that only express mouse tau. The migration profile revealed by DA9 showed a distinct band migrating at ∼52 kDa only in cultures that expressed mouse tau. This band was absent in non-transgene expressing control samples from *Mapt^0/0^* neurospheres indicating that it was 3R mouse tau, the only tau isoform expressed in mice during development [34]. Compared to non-transgene expressing controls (shown here: Tg(CK-tTA) and TRE-tau_P301L_), rTg samples had additional DA9 signals that appeared as a dense series of diffuse bands up to ∼60 kDa in rTg(tau_wt_) samples and ∼58 kDa in rTg(tau_P301L_) samples. Strikingly, the diffuse band in tau_wt_-expressing samples had a slower migrating component than tau_P301L_-expressing neurospheres. The slower migration of tau_wt_ also was revealed by DA9, Tau13, CP13, AT8, and PHF-1 antibodies (Figure 4A), possibly suggesting that wild type, rather than P310L mutant, tau was hyperphosphorylated. Immunostaining with an additional antibody, MC6, which is specific for pSer235, demonstrated that wt tau is more heavily phosphorylated at this epitope than P301L mutant tau; these results are presented in Figure S4.

To better characterize the tau species within the diffuse bands and the nature of the slower migrating tau_wt_ band, we used human-specific Tau13, phospho-tau specific antibodies, and alkaline phosphatase treatment. The diffuse series of bands in rTg samples and the slower migrating tau_wt_ species were observed with the Tau13 antibody confirming that they contained transgene-encoded human tau. All tau transgene-negative control samples lacked Tau13 signal confirming its specificity for human tau. We analyzed the neurosphere lysates for phosphorylated tau epitopes using CP13, AT8, and PHF-1 antibodies. The phospho-tau immunoreactivity in Tg(CK-tTA) and TRE-tau_P301L_ control samples indicated endogenous mouse tau and appeared as distinct 52 and 53 kDa bands (Figure 4A). Human transgene expressing samples with mouse tau contained the 52 and 53 kDa bands with an additional protein smear up to ∼60 kDa in rTg(tau_wt_) samples and to ∼58 kDa in rTg(tau_P301L_) samples indicative of phosphorylated human tau. Strikingly, the more slowly migrating tau_wt_ band became more apparent with the phospho-tau antibodies providing evidence that it was a phosphorylated tau species. Densitometric normalization to GAPDH, showed that rTg samples had greater immunoreactivity to CP13 (2.9x), AT8 (1.9x), and PHF1 (2.4x) than controls without human tau transgenes.

Tau1 immunoreactivity and alkaline phosphatase treatment confirmed that the migration difference between tau_wt_ and tau_P301L_ was due to phosphorylation. The Tau1 antibody recognizes tau species that are not phosphorylated at or near Ser^198/202^, and has decreased immunoreactivity when tau is phosphorylated at these epitopes. Both tau_wt_ and tau_P301L_-expressing cultures contained Tau1-immunoreactive non-phosphorylated tau with identical banding patterns migrating at ∼53 and 52 kDa (Figure 4A). TRE and Tg(CK-tTA) samples displayed only the 52 kDa band with Tau1 representing non-phosphorylated mouse tau. We treated lysates with calf intestinal phosphatase (CIP); to restrict analysis to human tau only, we analyzed neurosphere lysates from tau_wt_ and tau_P301L_ expressing cultures on the *Mapt^0/0^* background lacking endogenous mouse tau expression. Phosphatase treatment abolished the higher molecular weight bands (>52 kDa) providing evidence that they were phosphorylated human tau. Only a single tau band migrating at ∼52kDa, the known molecular weight of recombinant human tau 4R0N [35], remained after phosphatase treatment in both mutant and wild type samples (Figure 4B). Collectively, these data indicated that the difference between mutant and wild type tau protein migration was due to phosphorylation, and not other post-translational modifications. We did not recover sarkosyl insoluble tau indicating that insoluble tau aggregates did not form in undifferentiated cultures of either genotype indicating that tau hyperphosphorylation at tauopathy-associated epitopes such as those recognized by AT8 and PHF1 does not necessarily lead to tau aggregation (data not shown).

*Mapt^0/0^* cultures have not been fully characterized. Here we used them to help distinguish between mouse and human tau bands. The wt_3_ rTg(tau_wt_) *Mapt^0/0^* culture with low total tau (DA9) and human tau (Tau13) immunoreactivity may be due to the low proportion of human tau expressing cells (42%) compared to the other rTg(tau_wt_) *Mapt^0/0^* culture, wt_4_ with 62% tau-expressing cells.

Brain homogenates from E14, the developmental stage at which neurospheres were harvested, and young adult 2.5 month-old mice were analyzed for tau phosphorylation. As seen in neurospheres, rTg(tau_wt_) brain homogenates displayed a distinctly slower migrating tau species than rTg(tau_P301L_) (Figure 4C) demonstrating that neurospheres reliably model the difference between wild type and mutant tau phosphorylation that is seen *in vivo*. Of note, a lower molecular weight mouse tau isoform that stained intensely with phospho-tau antibodies in E14 brains was absent in 2.5-month-old mice. During embryonic development, the vast majority of tau is the lower molecular weight 3R tau isoform (∼48 kDa without posttranslational modifications) [34] that is heavily phosphorylated [36], [37]. By postnatal day 24, fetal tau isoforms have been replaced with larger, though less phosphorylated, tau isoforms [34], [38]. The lower molecular weight tau species unique to E14 brains likely represents heavily phosphorylated fetal mouse tau. Also of note, adult rTg(tau_wt_) mice contained a higher molecular weight Tau1-immunoreactive tau species than adult rTg(tau_P301L_), suggesting that young adult rTg(tau_wt_) mice express a tau species not phosphorylated at Ser^198/202^ that is either more heavily phosphorylated at other epitope(s), or has other post-translational modifications distinct from tau_P301L_. Results from these analyses are summarized in Table 1.

**Figure 5:**
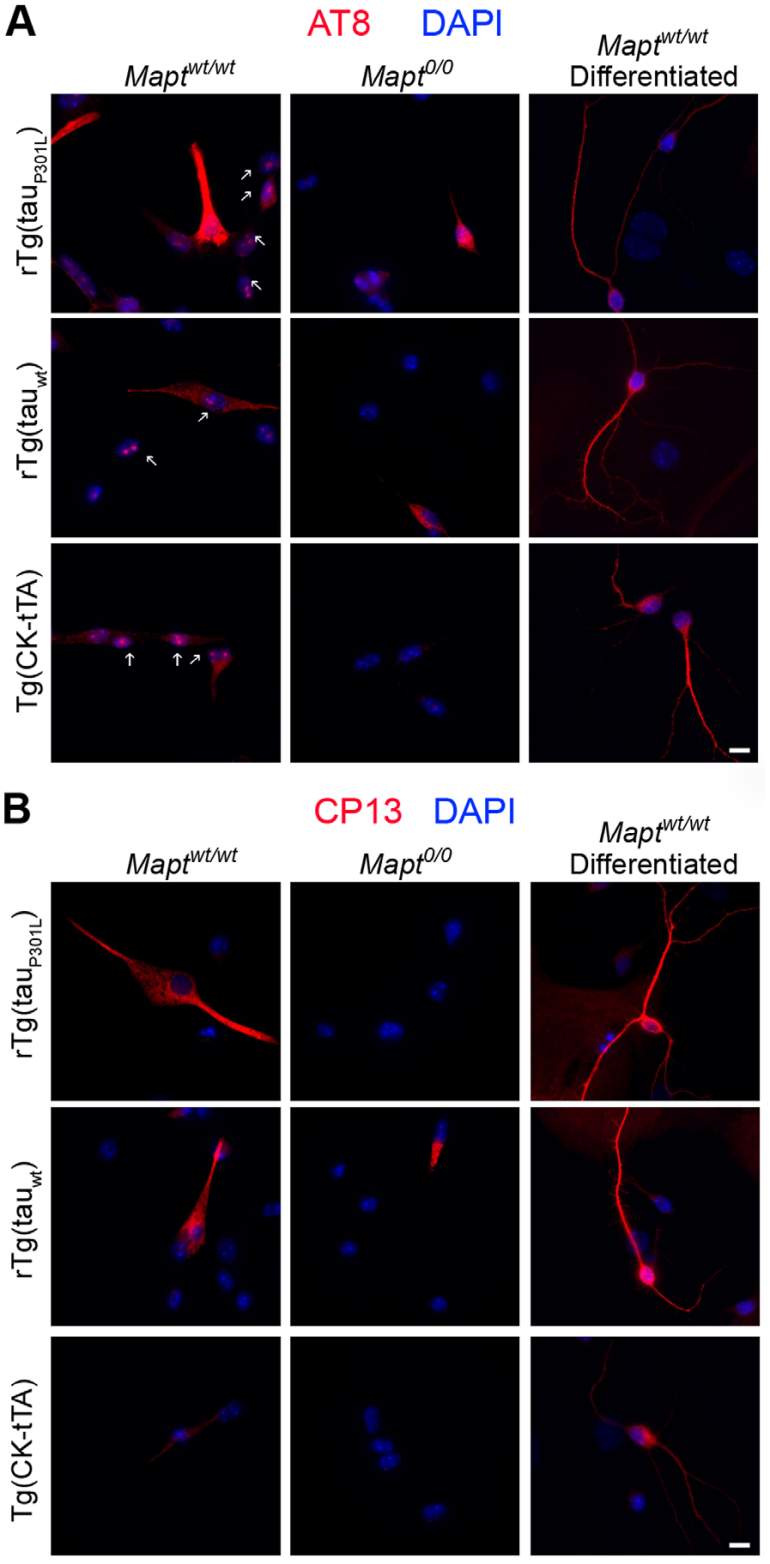
Overexpression of human tau did not interfere with localization of mouse tau to the nucleus in undifferentiated dividing cells. IFA revealed a punctate nuclear tau species (arrow) immunoreactive to AT8 (A) but not CP13 (B) in dissociated undifferentiated neurospheres. AT8 immunoreactivity, indicative of pSer^202^/Thr^205^ tau, was seen in the nucleus of all genotypes that expressed mouse tau, but was absent in cultures derived from *Mapt^0/0^* or in differentiated cells providing evidenced that it was of mouse origin and corresponded to actively dividing cells but not mature cell types.

We assessed tau phosphorylation in dispersed cells from dissociated neurospheres. For each cell culture we determined the proportion of nestin stained cells that also were immunoreactive with phospho-tau antibodies. rTg cultures contained a greater proportion of cells immunoreactive to phospho-tau antibodies than Tg(CK-tTA) controls. The AT8 antibody revealed focal staining in nuclei of all genotypes expressing mouse tau (Figure 5). The staining was absent in *Mapt^0/0^* cells and likely represents binding of 3R pSer^202^/Thr^205^ mouse tau to nucleolar organizing regions in dividing cells as reported previously [39]–[41]. This staining was not observed with CP13, which recognizes a different epitope associated with pSer^202^, nor in non-dividing cells after differentiation. The presence of appropriately phosphorylated nucleolar mouse tau in dividing, but not differentiated cells, indicates that overexpression of human tau did not compromise physiological tau modification and function.

### Stem or Progenitor Cell Populations that Express Human Tau Transgenes Maintained Stem Cell Properties

We examined the variability of self-renewal and differentiation among independent cell lines. Although there was an effect of the P301L mutation on tau phosphorylation, expression of mutant or wild type human tau did not obviously interfere with their SC properties. Human tau expression did not alter the proliferation rate of neurosphere lines; regardless of genotype, all cell lines required passaging every 5–7 days. We conducted quantitative cell proliferation assays between passages 2–3 and between passages 6–7 on cell lines of all genotypes. Independent cultures of all genotypes had similar doubling times ranging from 20 to 32 hours as previously reported for neural SC lines [42]. Tau_P301L_-expressing cultures had a shorter, though non-significant, doubling time of 23.3±4 hours (n = 3), than controls and rTg(tau_wt_) with 29.4±8.8 hours (n = 5) and 29.3±5.5 hours (n = 3) doubling times. Sex of the mice of neurosphere origin did not influence transgene expression or growth rates.

**Figure 6:**
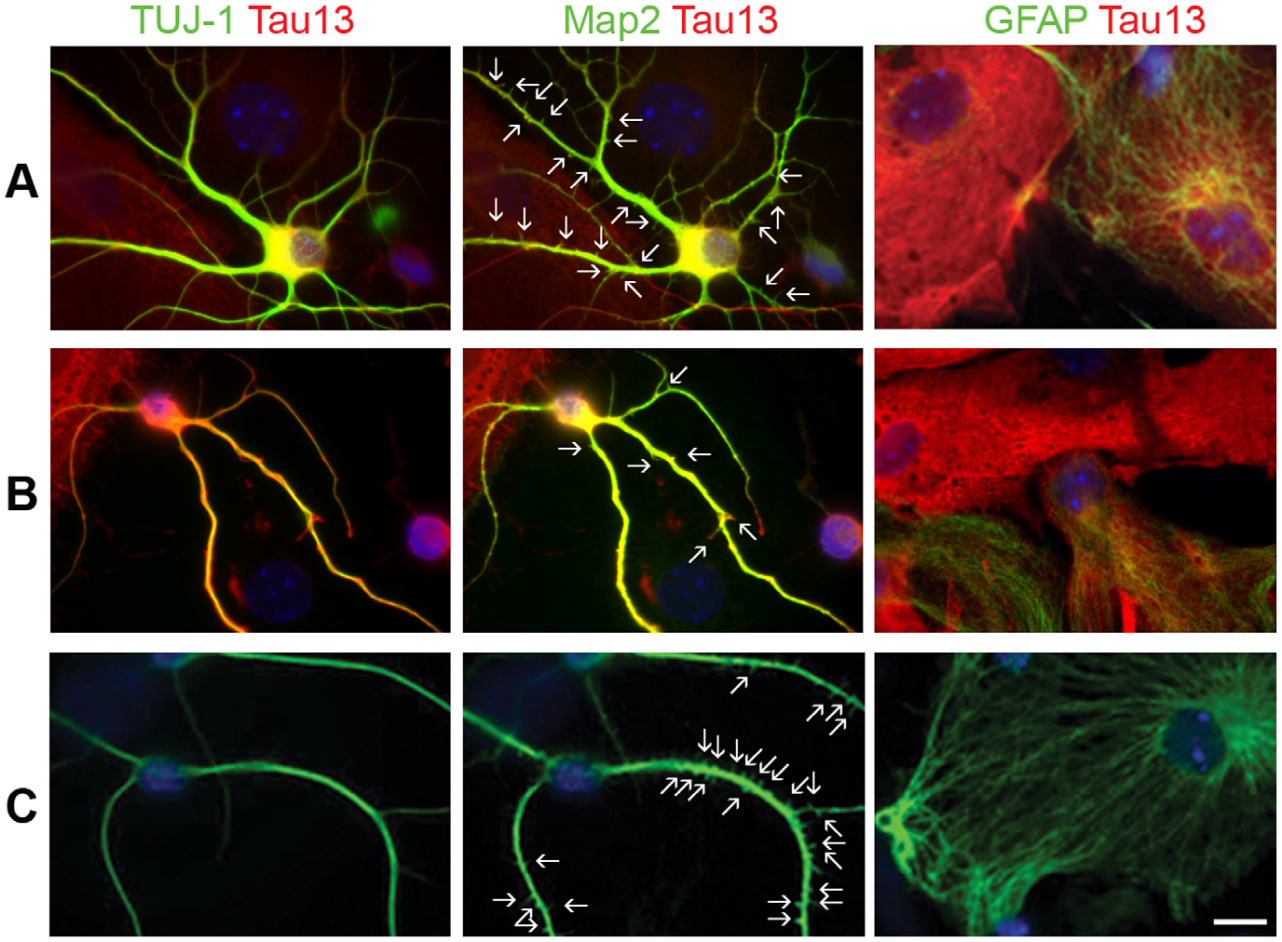
Dissociated neurospheres stimulated with retinoic acid differentiated into cells that expressed neuronal or glial antigens. Each rTg(tau_P301L_) (panel A) and rTg(tau_wt_) (panel B) culture contained cells positive for Tau13 and TUJ-1, Tau13 and Map2, and Tau13 and GFAP; merged images of Tau13 (red) with TUJ-1, Map2, or GFAP (green) are presented; yellow color indicates overlapping expression. Tg(CK-tTA) control cells are shown in panel C. Arrows point to spiny projections labeled with Map2 staining. Scale bar  = 10 µm. Cells were simultaneously stained with three different secondary antibodies (Alexa 546 α-mouse for Tau13; Alexa 647 α-rabbit for TUJ-1; Alexa 488 α-chicken for Map2) and each fluorophore was imaged in separate channels. Alexa 546 was artificially colored red, and Alexa 647 and 488 were both artificially colored green for color-combine with Tau13.

To investigate effects of transgene expression on neurosphere multipotency and differentiation, we plated dissociated neurosphere cells on laminin/poly-L-ornithine coated glass bottomed plates in basal medium supplemented with retinoic acid [43], [44]. After 21 or 25 days, we fixed, stained, and visualized the cells with IFA using antibodies against neuronal and glial antigens. Differentiated rTg(tau_P301L_), rTg(tau_wt_), and Tg(CK-tTA) control neurospheres all contained cells positive for neuronal proteins microtubule associated protein 2 (recognized by Map2 antibody) and β-tubulin III (recognized by TUJ-1 antibody) or the astrocytic protein, glial fibrillary acidic protein (GFAP) (Figure 6). Though mature neurons restrict Map2 expression to dendritic spines, immature neurons express Map2 in developing axons as well as in developing dendrites [45]–[48]. Neuritic projections contained both β-tubulin III and Map2 expression, but in contrast to the smooth axonal TUJ-1 staining, Map2 staining had a spiny morphology indicative of developing dendritic-spines (Figure 6).

**Figure 7:**
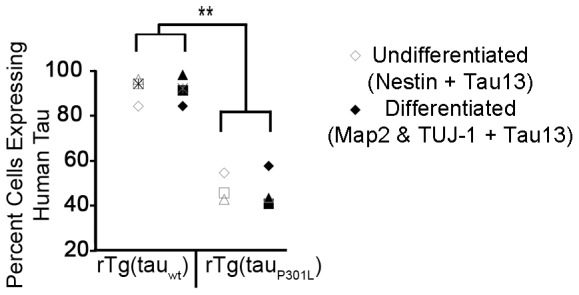
The proportion of differentiated cells that expressed human tau reflected that of undifferentiated neurospheres. Differentiated cells immunoreactive with both Map2 and TUJ-1 antibodies were counted; the proportion co-expressing Tau13 is presented here. Presented are results from four independent rTg(tau_wt_) cultures and three independent rTg(tau_P301L_) cultures generated from two litters of each Tg(CK-tTA) × TRE-tau mating. The four litters were harvested, genotyped, and cultured simultaneous. They yielded four rTg(tau_wt_) and three rTg(tau_P301L_) pups for neurosphere culture shown here. By ANOVA, the proportions of differentiated cells (closed symbols) did not differ from those of the neurospheres (open symbols) (ANOVA, F = .008, P = .93). The different symbols represent individual cell lines before and after differentiation. Genotype had a highly significant effect even after Bonferroni correction (**: ANOVA, p<0.0001).

The rTg cell lines contained cells that co-expressed neuronal proteins and human tau and cells that co-expressed GFAP and human tau (Figure 6A, B). All genotypes contained similar proportions of cells immunoreactive with both Map2 and TUJ-1, 3–5%, indicating that transgene expression did not alter neuronal cell differentiation. Compared to tau_wt_-expressing cultures, tau_P301L_ cultures contained fewer neuronal protein expressing cells that also expressed human tau; the proportions mirrored that of undifferentiated cells co-expressing nestin and human tau (Figure 7).

**Figure 8:**
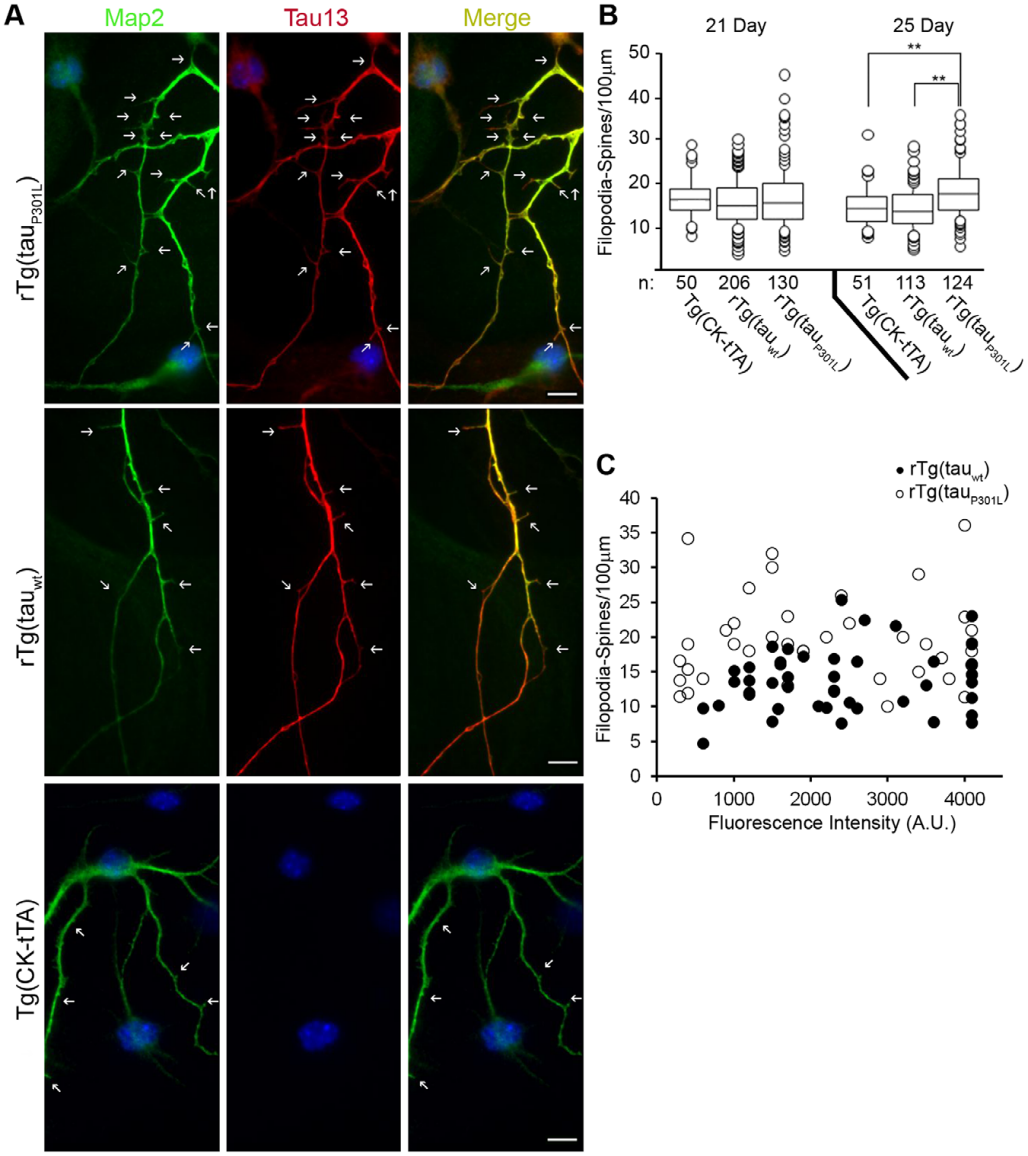
rTg(tau_P301L_) cells developed more filopodia-spines than differentiated rTg(tau_wt_) and non-transgene expressing control cells. (A) Cells differentiated for 21 or 25 days were evaluated for the number of Map2 positive filopodia-spines and tau localization. Shown here are data pooled from three independent rTg(tau_P301L_) and four independent rTg(tau_wt_) lines. We observed transgenic tau expression, visualized by Tau13 immunoreactivity, throughout Map2 positive neurites and filopodia-spines in both tau_P301L_ (top panel) and tau_wt_ (bottom panel) expressing cells. Arrows point to filopodia-spines. Scale bar  = 10 µm. (B) The density, filopodia-spines/100 µm, was plotted for each genotype (n indicates number of cells counted). Time post-differentiation did not significantly affect spine density (ANOVA, F = 1.2, P = .28), but there was a significant interaction between genotype and time (F = 6.5, P = 0.0016) with spine density in rTg(tau_P301L_) neurospheres at 25 days significantly (**, P<.0001) greater than in rTg(tau_wt_) or TgCKtTA neurospheres. Overall the effect of genotype was highly significant (ANOVA, F = 13.3, P<0.001) The horizontal lines inside the boxes demarcate the mean filopodia-spine densities, circles indicate outliers; “n” indicates number of cells counted. (c) Plotting of spine density against fluorescence intensity revealed no correlation (R^2^ = 0.01) between protein expression level and filopodia-spine density. Closed circles represent rTg(tau_wt_) cells, open circles represent rTg(tau_P301L_) cells.

We observed a difference in filopodia-spine density between rTg(tau_P301L_) and rTg(tau_wt_) differentiated cells at 25 days after exposure to differentiation stimuli. Mature dendritic spines develop from elongated filopodial projections [49]–[53]. Here we counted the density of these dendritic spine precursors, filopodia-spines, from differentiated cells derived from neurosphere cultures. The filopodia-spine density among genotypes did not differ significantly at 21 days post-differentiation, but became statistically significant 4 days later (Figure 8B). The filopodia-spine density increased between days 21 and 25 in tau_P301L_ cultures; conversely, the filopodia-spine density in the tau_wt_ and control cultures slightly decreased during this interval. At 25 days post-differentiation, cells derived from tau_P301L_-expressing neurospheres had a 23±3% higher filopodia-spine density than neurons derived from tau_wt_-expressing cultures, and 20+/−4% higher filopodia-spine density than non-Tg expressing controls (p<0.0001); rTg(tau_P301L_) *Mapt^0/0^* and rTg(tau_wt_) *Mapt^0/0^* cultures showed similar results (data not shown). Interestingly, we saw an association with cell culture genotype, but not with the level of transgene expression in individual cells, with spine density. Within a single culture, cells expressing high levels of human tau had filopodia-spine densities similar to those in cells expressing low levels of human tau and cells in which transgene-encoded protein was not detectable (Figure 8C). We did not observe any genotype-specific differences in tau localization to spines in differentiated cells (Figure 8A); transgene-positive tau_wt_ and tau_P301L_ cells expressed human tau throughout their neuritic projections.

In addition to cell types with antigen expression and morphology characteristic of neurons or astrocytes, a population of very large cells that, in some cases, had a diameter over 100 µm emerged. These cells had a flattened appearance, some were elongated, and they expressed low levels of nestin and GFAP but not Map2, β-tubulin III, or GABA; some expressed human tau. These cells first appeared in culture ∼7 days after induction of differentiation and grew in size until the cultures reached confluence. Examples of these cells can be seen in Figure 6. They appeared after growth factor withdrawal upon receiving differentiation stimuli and developed morphological phenotypes resembling senescent cells [54] and may indicate cells that did not receive the appropriate stimuli to complete differentiation and remained in a pseudo-stem cell state. While morphologically and antigenically distinct neural precursor cells have been identified in neurospheres, one of which is EGF-responsive and the other morphologically large EGF/FGF-2 responsive cells [55], we did not supplement cultures with FGF-2. Though neurospheres may have endogenously synthesized FGF-2 [25], the uncharacterized cells did not display strong nestin immunoreactivity typical of neural SCs.

## Discussion

The impetus for these studies was to use a well-characterized mouse model for frontotemporal dementia to assess whether CNS-SC cultures reproduced genetic differences seen in the mice from which they were derived and whether independent isolates from genetically identical hosts produced consistent phenotypes. Neurospheres derived from tau_wt_–expressing mice contained more heavily phosphorylated tau species with slower electrophoretic motility than tau_P301L_. The phosphorylation differences between tau_P301L_ and tau_wt_ also occurred in fetal mouse brains and persisted through young adulthood. Though hyperphosphorylated tau is considered a hallmark feature of tauopathy, several disease-causing mutant tau variants actually are less phosphorylated than tau_wt_ *in vitro* [56]–[59] and in young mice [60]–[62]. While abnormally phosphorylated tau in aged rTg(tau_P301L_)4510 mice coincides with memory and behavioral abnormalities [20], [60], the hypophosphorylated tau_P301L_ seen in young mice may have pre-clinical significance and deserves attention. Transgenic mice that express pseudohyperphosphorylated tau, an engineered tau variant that mimics constitutively phosphorylated tau by replacing Ser/Thr residues with glutamate [63], did not develop tau aggregates, neuronal loss, or behavioral abnormalities [64]. In contrast, mice expressing tau_R406W_, a variant that remains hypophosphorylated compared to tau_wt_ even in aged mice, developed age-dependent tau aggregates [65] and memory and behavioral abnormalities [66].

The hypophosphorylated tau_P301L_ species in neurospheres and young mice may represent free tau not associated with microtubules (MT). Tau bound to MTs acquires more phosphorylation than free tau as demonstrated by *in vitro* phosphorylation of tau in the presence or absence of MTs [59], [67]. Many of the MAPT exon 10 missense mutations that cause dementia, including P301L, reduce the ability of tau to interact with MT [68]–[71], and tau_wt_ displaces mutant tau from MTs [63], [72]. The absence of tau_P301L_ aggregates or neurofibrillary tangles (NFTs) in neurospheres and in young mice, despite phosphorylation at many of the sites most frequently phosphorylated in AD and FTD (pSer^202^, pSer^396^, pSer^235^, pSer^404^) [36], [73]–[76], also may correspond to unbound tau as MT association has been implicated as an important step for tau nucleation [77], [78]. The quantity of NFTs correlates with disease severity [79]. However, recent studies have dissociated NFTs from neuronal death [80]–[82] and decreased memory function [19], and instead suggest a deleterious effect of soluble tau.

Phosphorylation of mouse tau also reflects appropriate phosphorylation corresponding to the differentiation state. Heavily phosphorylated 3R mouse tau is know to bind to nucleolar organizing regions in dividing cells [39]–[41] and was also observed in neurospheres, indicating that human tau did not inhibit either the cellular machinery or kinases involved. This reinforces the same conclusion coming from the recapitulation of the genetic differences in human tau phosphorylation seen in neurosphere culture.

We observed differences in filopodia-spine densities between tau_wt_ and tau_P301L_ differentiated cells. Developmentally, dendritic spine morphology evolves from long thin filopodia-spines to mature spines of various morphologies; during this transformation, filopodia-spine density decreases [49]–[53]. We observed a slightly higher filopodia-spine density in Map2 and TUJ-1 double-positive cells from tau_P301L_ cultures than those derived from tau_wt_ or cultures that did not express MAPT. While the level of transgene expression within individual cells did not affect filopodia-spine density, we have not ruled out an effect of the transgene insertion site. Interestingly, in a separate P301L mouse line that that harbors the mutation in the longest mouse tau isoform, 4R2N, driven by the *Thy1* promoter, young mice with “hypophosphorylated” tau have enhanced learning and memory and increased Long Term Potentiation (LTP) in the dentate gyrus compared to controls [61]. With age, the spine density of rTg(tau_P301L_)4510 mice decreases and coincides with increased neuronal excitability [81]. Whether or not the greater filopodia-spine density we observed in differentiated tau_P301L_ cultures relates to enhanced LTP in young mice or neuronal vulnerability later in life in unknown, but warrants further exploration.

We did not observe mislocalization of tau_P301L_ to dendritic spines, as reported in aging mice [60] or in transfected rat neuron cultures [21]. We saw high levels of transgenic tau protein throughout neuritic projections in differentiated neurospheres from both human tau transgene genotypes. The presence of tau in the filopodia-spines of developing cells [74], [83] suggests that the phenomenon correlates with a dynamic morphology and does not necessarily indicate pathological injury.

Overall, phenotypes of neurosphere cultures isolated from individual fetuses at different times reflected the genotype of the mice from which they were derived and proved highly similar in transgene expression, proliferation, and differentiation. Unlike clonal cultures, these neurospheres were formed by aggregation, which results in culture heterogeneity [22]–[25]. During culture they merge and fuse as well as proliferate [84], [85], which likely resulted in the heterogeneous composition of transgene expressing and non-expressing cells within the same sphere. The minor transgene expression variability among neurosphere cultures generated from littermate fetuses possibly occurred during the initial brain harvest. We did not specifically dissect the forebrain from each fetus. Nestin expressing neural SCs in the developing midbrain [86] and hindbrain [87] may have contributed to the transgene expressing cell population since CKIIα, the promoter driving tTA-transgenic human tau expression, is expressed throughout the brain at this developmental age [88]. We saw more variability among independent experimental harvests than among cultures derived from littermate fetuses; we attribute this variation to inconsistencies inherent to IFA. Regardless, IFA consistently demonstrated that undifferentiated cells derived from rTg(tau_wt_) expressing fetal brains expressed human tau in a higher proportion of cells than those derived from rTg(tau_P301L_) expressing fetal brains.

Total brain homogenates indicate that rTg(tau_wt_) mice express comparable levels of transgenic tau as rTg(tau_P301L_) [21]. While the rTg(tau_P301L_) cultures had a lower proportion of total cells expressing human tau, they contained a greater proportion of cells that expressed higher levels of tau (brighter fluorescence intensity). This feature of some transgenes is caused by position-effect variegation [89]–[91]. Neurospheres, like the mice from which they were derived, may show this effect and could be useful models for screening transgene expression in founder lines. Consistent with the difference in transgene expression seen in undifferentiated neurospheres, differentiated cells derived from tau_wt_-expressing neurospheres expressed human tau in a higher proportion of cells than those derived from tau_P301L_-expressing neurospheres. Likely, non-transgene expressing progenitor cells gave rise to non-transgene expressing differentiated cells and transgene expressing progenitor cells differentiated into transgene expressing mature cells. Alternatively, tau_P301L_ transgene expression may have decreased neural precursor survival. Since neurospheres proliferated and differentiated over several passages in both genotypes, and differentiated transgene expressing cell proportions mirrored that of the undifferentiated condition, evidence favors the former explanation.

Some of the differences we saw between tau_P301L_ and tau_wt_ may stem from differences in transgene insertion sites. Neurosphere cultures from mouse lines expressing the same human tau variants, but at lower levels than rTg(tau_P301L_)4510 and rTg(tau_wt_)21221, overlapped in the percentages of cells expressing human tau, but within the same range as our more extensively studies lines. It is likely that there are effects of transgene insertion site as well as the tau mutation.

With the emergence of methodologies to culture neurospheres from human patients [14], experiments evaluating the culture system’s relevance and validity are crucial. Our data provide supporting evidence that neurospheres can reliably model phenotypes of their derived source over extended culture periods. The neurosphere culture system provides a robust assay for studying effects of external factors on the development and differentiation of the CNS, and the genetic susceptibility to neurological disorders. While it is unreasonable to expect that an *in vitro* system will fully recapitulate a complex disease process involving complex cell interactions, biologically relevant models that provide reproducible results are invaluable resources. This SC model shares the genetics of mice that model human FTD, and has shown the capacity to model pre-disease, genotype-specific phenotypes. By using mice expressing either of two distinct transgenes as the source of SCs, we have established that neurosphere cultures maintain genotype-specific characteristics.

Our results lend credence to the growing body of data supporting the development and use of patient specific-stem cell lines to study disease. We have already shown that these cells reproducibly mimic biological events of the mice from which they were derived, and that they express the appropriate molecules involved in tau modification genetically validating the utility of SC as a model system. We are now in a position to interrogate the system. We are focusing on microarray analysis experiments to uncover differentially regulated genes between tau_P301L_ and tau_wt_ mice and neurosphere cultures. Preliminary results are encouraging and show consistency in genotype-specific gene expression patterns among independently derived neurosphere lines. This will direct hypotheses about potential pathways targeted in cells that carry the tau_P301L_ gene. By extending this research to patient-specific SCs, high-throughput cell based genetic screening (cDNA or siRNA) assays could uncover small molecules and potential pathways involved in pathogenesis and, ideally, the genetic specificity of the system may lead to treatment therapies tailored to unique patient needs.

## Supporting Information

Figure S1 **Entire, unrearranged immunoblots used to generate** **Figure 4** **.** Figure 4 immunoblots were rearranged for clarity of presentation and some lanes removed for uniform presentation of our results. In this supporting figure, we present entire blots with their respective GAPDH loading controls. (A) Lane loading was identical for DA9 and Tau1 membranes as shown. (B) The Tau13 blot was loaded in the same orientation as DA9 and Tau1, but contained only the TRE_wt_ control. (C) Lane arrangement was identical for CP13 and AT8 membranes. (D) Lane arrangement for PHF-1. In Figure S1 A–D, DA9, Tau1, Tau13, and PHF-1 membranes were blotted simultaneously with GAPDH and the full membranes are shown. CP13 and AT8 antibodies required a more sensitive ECL system than the other antibodies, requiring probing GAPDH independently after cutting the blot; membrane reconstruction is shown. rTg(tau) sample labels (i.e. wt_1_, P301L_1_, wt_2_, P301L_2_, etc.) correspond to those shown in Figure 4.
(TIF)

Figure S2 **Original immunoblots used to generate** **Figure 4C** **showing results from embryonic day 14 mice.** For simplicity, only one representative mouse for each genotype was shown in Figure 4C; all samples are shown here. GAPDH was probed simultaneously with each of the anti-tau antibodies; whole immunoblots are shown.
(TIF)

Figure S3 **Original 2.5-month-old mouse immunoblots used to generate** **Figure 4C** **.** For simplicity, only one representative mouse for each genotype was shown in Figure 4C; all samples are shown here. GAPDH was probed simultaneously with each of the anti-tau antibodies; whole immunoblots are shown.
(TIF)

Figure S4 **rTg(tau_wt_) neurospheres were more heavily phosphorylated at Ser^235^ than rTg(tau_P301L_) neurospheres.** rTg(tau_wt_) and rTg(tau_P301L_) neurospheres, with or without endogenous mouse tau, were immunoreactive with the MC6 (anti-pSer^235^) antibody. As seen with the other phospho-tau antibodies, rTg(tau_wt_) neurospheres displayed a slower migrating band than rTg(tau_P301L_) neurospheres. The membrane was subsequently probed with Tau13; the characteristic migration difference between tau_wt_ and tau_P301L_–expressing samples was apparent.
(TIF)
