# Supplementary figures and images for: Genotype-Specific Differences between Mouse CNS Stem Cell Lines Expressing Frontotemporal Dementia Mutant or Wild Type Human Tau
Source: PLoS One. 2012 Jun 18;7(6):e39328. doi: 10.1371/journal.pone.0039328 (PMC3377636; doi:10.1371/journal.pone.0039328)

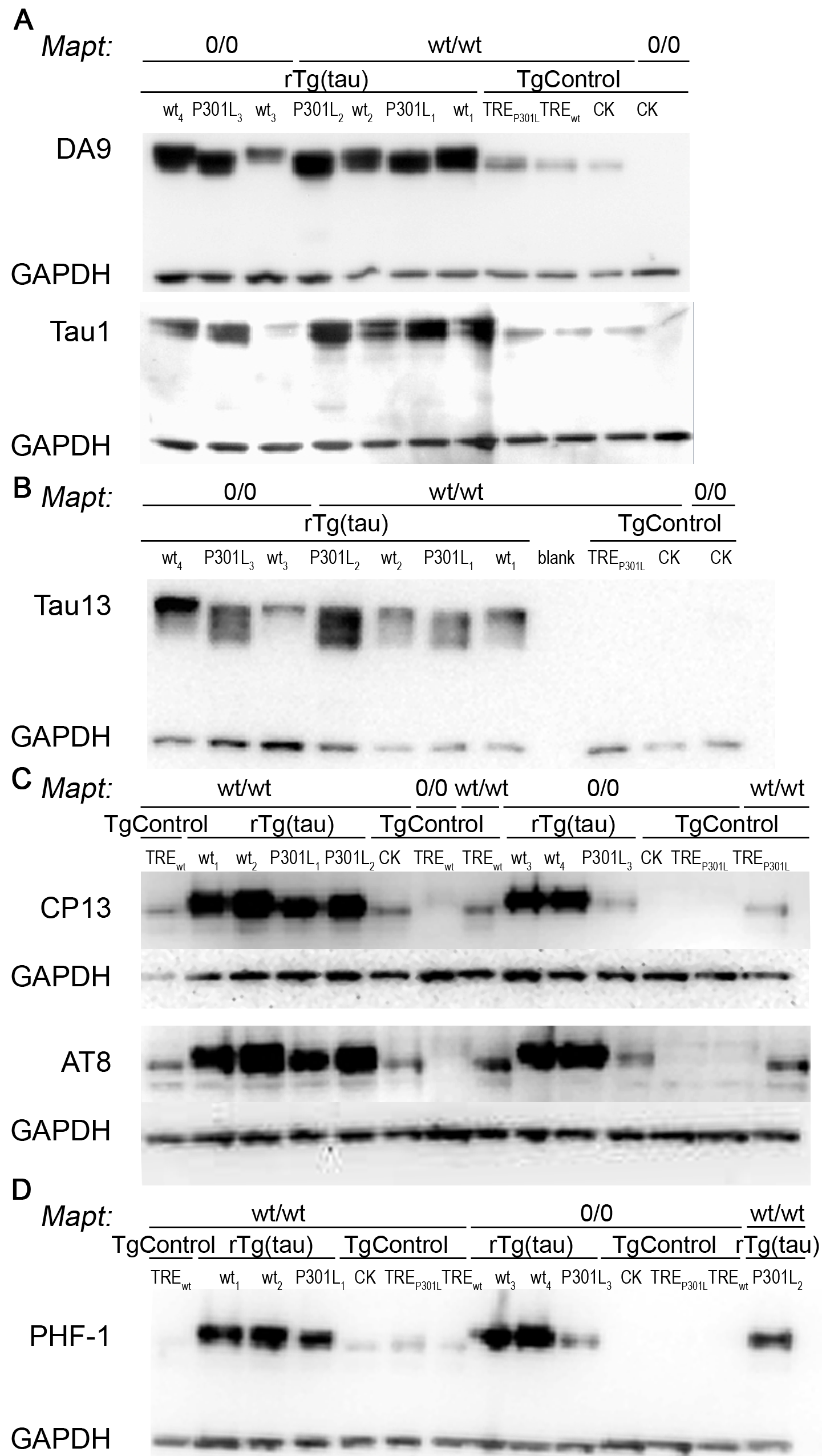

Supplement: Figure S1 — Entire, unrearranged immunoblots used to generate Figure 4 . Figure 4 immunoblots were rearranged for clarity of presentation and some lanes removed for uniform presentation of our results. In this supporting figure, we present entire blots with their respective GAPDH loading controls. (A) Lane loading was identical for DA9 and Tau1 membranes as shown. (B) The Tau13 blot was loaded in the same orientation as DA9 and Tau1, but contained only the TREwt control. (C) Lane arrangement was identical for CP13 and AT8 membranes. (D) Lane arrangement for PHF-1. In Figure S1 A–D, DA9, Tau1, Tau13, and PHF-1 membranes were blotted simultaneously with GAPDH and the full membranes are shown. CP13 and AT8 antibodies required a more sensitive ECL system than the other antibodies, requiring probing GAPDH independently after cutting the blot; membrane reconstruction is shown. rTg(tau) sample labels (i.e. wt1, P301L1, wt2, P301L2, etc.) correspond to those shown in Figure 4. (TIF) [file pone.0039328.s001.tif]

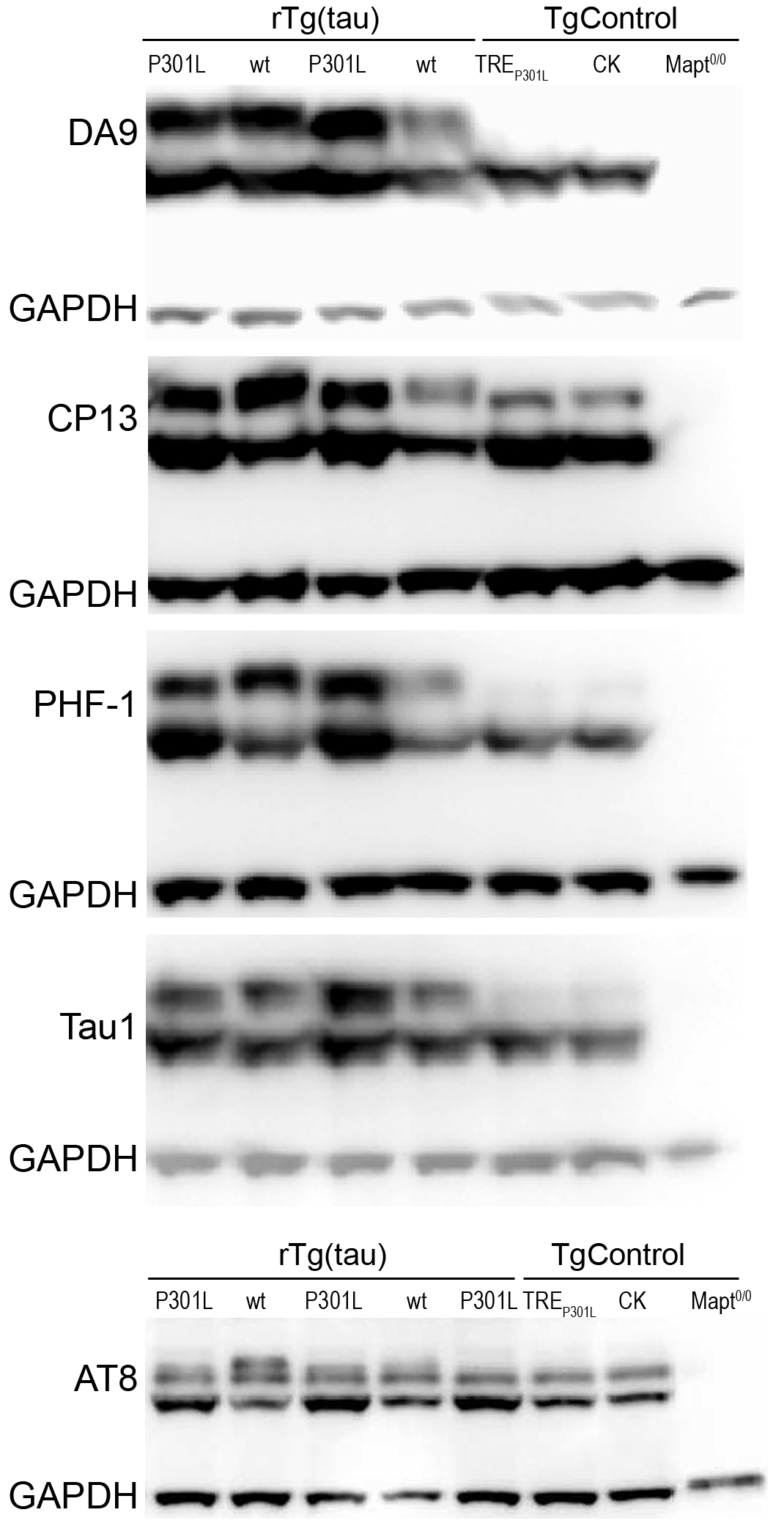

Supplement: Figure S2 — Original immunoblots used to generate Figure 4C showing results from embryonic day 14 mice. For simplicity, only one representative mouse for each genotype was shown in Figure 4C; all samples are shown here. GAPDH was probed simultaneously with each of the anti-tau antibodies; whole immunoblots are shown. (TIF) [file pone.0039328.s002.tif]

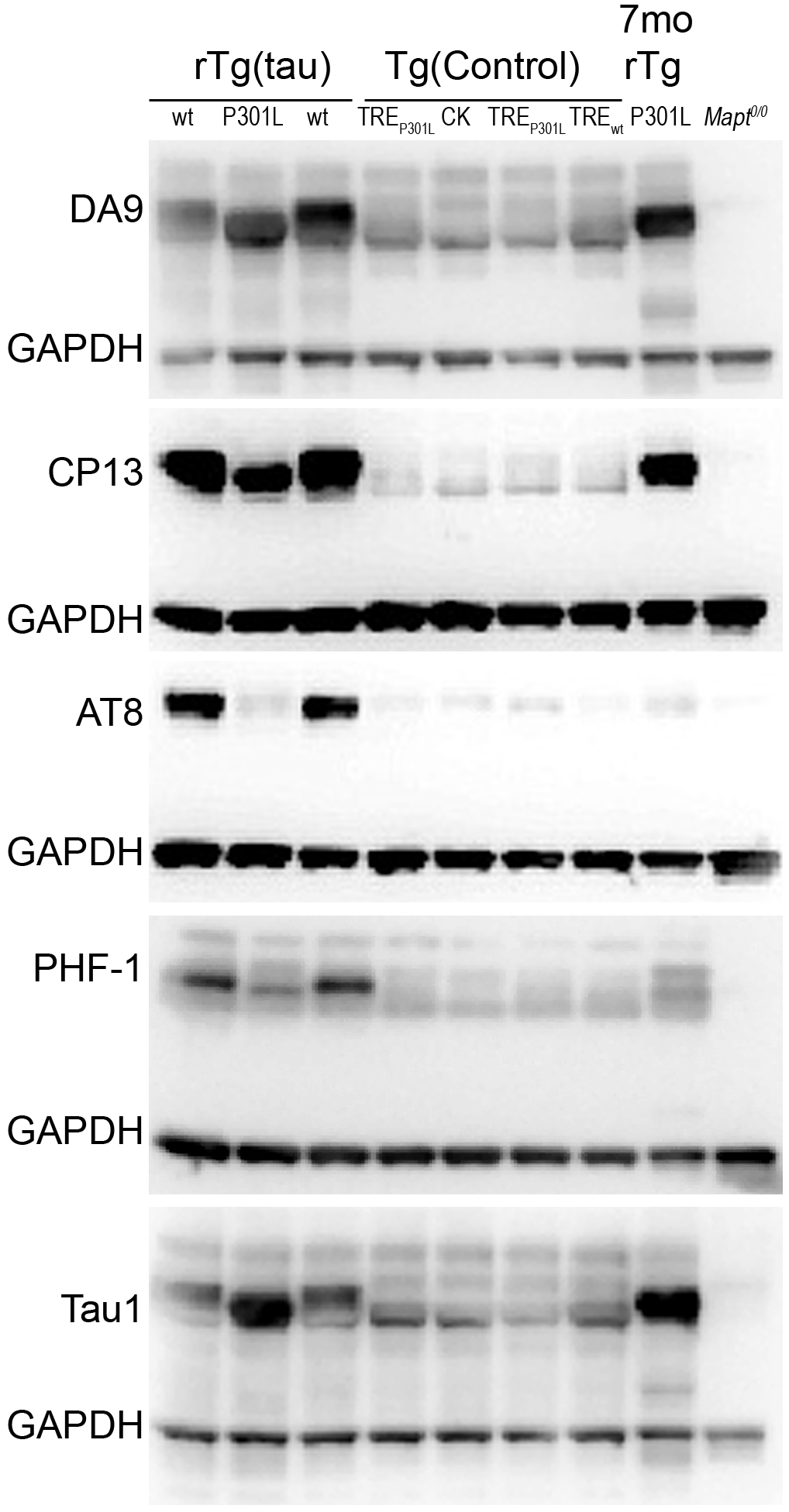

Supplement: Figure S3 — Original 2.5-month-old mouse immunoblots used to generate Figure 4C . For simplicity, only one representative mouse for each genotype was shown in Figure 4C; all samples are shown here. GAPDH was probed simultaneously with each of the anti-tau antibodies; whole immunoblots are shown. (TIF) [file pone.0039328.s003.tif]

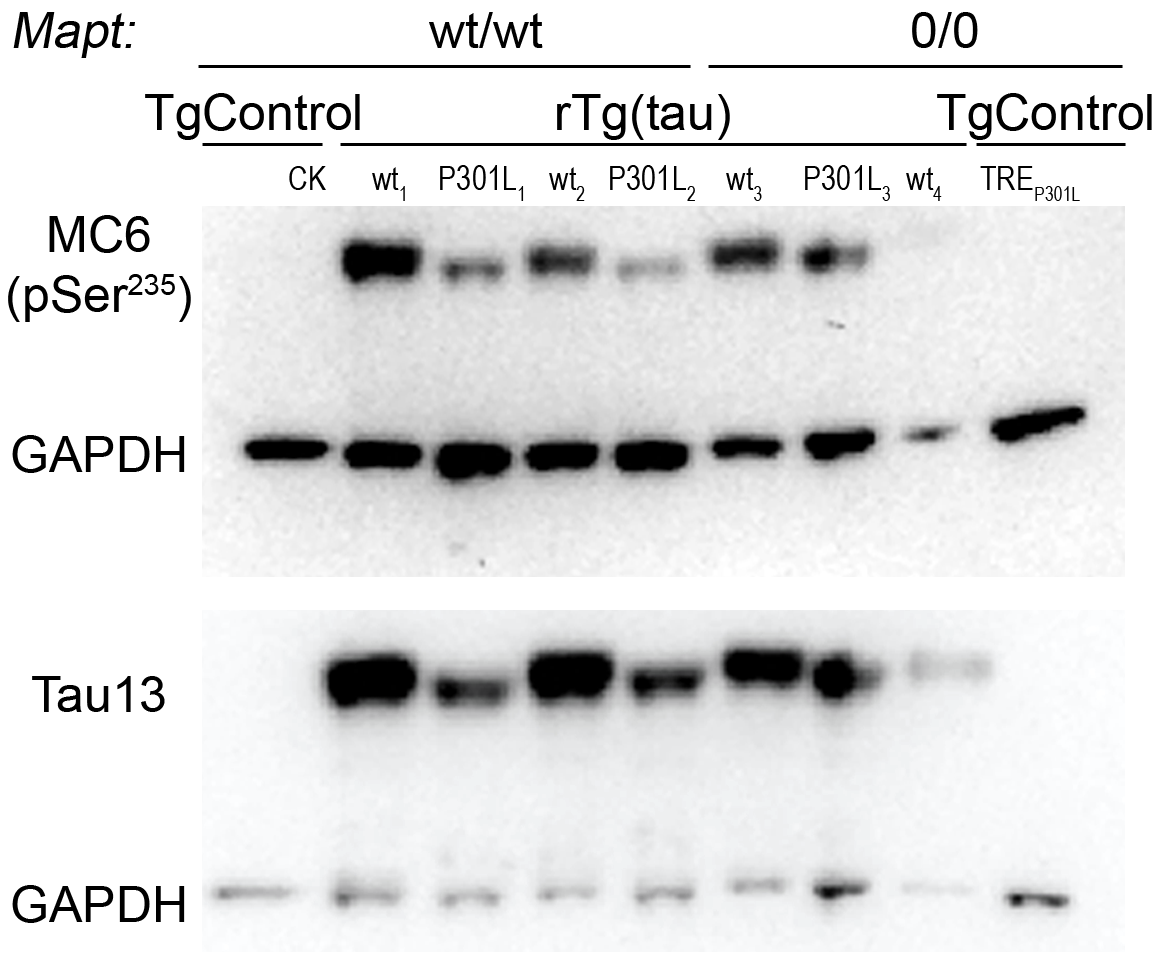

Supplement: Figure S4 — rTg(tauwt) neurospheres were more heavily phosphorylated at Ser235 than rTg(tauP301L) neurospheres. rTg(tauwt) and rTg(tauP301L) neurospheres, with or without endogenous mouse tau, were immunoreactive with the MC6 (anti-pSer235) antibody. As seen with the other phospho-tau antibodies, rTg(tauwt) neurospheres displayed a slower migrating band than rTg(tauP301L) neurospheres. The membrane was subsequently probed with Tau13; the characteristic migration difference between tauwt and tauP301L–expressing samples was apparent. (TIF) [file pone.0039328.s004.tif]
